# Civilians under missile attack: post-traumatic stress disorder among the Jewish and Bedouin population of Southern Israel

**DOI:** 10.1186/s13584-024-00625-9

**Published:** 2024-08-12

**Authors:** Rachel Shvartsur, Bella Savitsky

**Affiliations:** Department of Nursing, School of Health Sciences, Ashkelon Academic College, Yitshak Ben Zvi 12, Ashkelon, Israel

**Keywords:** Armed conflict, Bedouins, Health disparity, Mental health, Posttraumatic stress disorder, War exposures

## Abstract

**Background:**

Over the past 20 years, Jewish and Bedouin civilians in southern Israel have faced the ongoing threat of missile attacks from Gaza, with possible mental health consequences. This study aimed to assess the prevalence of post-traumatic stress disorder (PTSD) among Jewish and Bedouin adult civilians in southern Israel in a period with few missile attacks from Gaza, and no military operations.

**Methods:**

The study population included 389 participants (246 Jews, 143 Bedouins) living within 40 km/25 mi from Gaza for at least 2 years and interviewed between January and March 2023 (before the ongoing war that started on October 7th, 2023). The PTSD Checklist (PCL-5) was used, with a score of 33 as a cutoff point for the presence of PTSD.

**Results:**

Compared to Jews, a significantly lower proportion of Bedouins reported accessibility to bomb shelters and siren warning systems. Overall, 20.3% of the respondents exhibited PTSD. Multivariate analysis revealed that after adjustment for demographic and household characteristics, Bedouins had a six-fold significantly higher probability of PTSD in comparison to Jews (OR 5.6, 95%CI 2.8–10.8). Compared to participants with high socioeconomic status (SES), participants with low SES had a six-fold significantly higher probability of PTSD (OR 6.0, 95%CI 2.2–16.5). Participants who did not have an alarm system had more than two-fold odds for PTSD (OR 2.3, 95%CI 1.1–5.5). Being single, living in urban areas, or having a disability significantly increased the probability of PTSD.

**Conclusions:**

The findings of this study demonstrate a significantly higher prevalence of PTSD among the Bedouin population of Southern Israel. Several sociodemographic characteristics were associated with the increased prevalence of PTSD, the most prominent of which was low SES. Healthcare professionals and authorities should be proactive in screening for PTSD, and provide tailored treatment and support, taking into account ethnical and cultural background. Authorities should address the disparity in bomb shelter access and siren warning coverage between Bedouin and Jewish communities.

## Background

In the last two decades, especially after Hamas took over the Gaza Strip in June 2007, civilians in southern Israel—both Jews and Bedouins—have been subjected to an ongoing threat of missile attacks by various armed groups. Throughout these years, missiles were launched at homes, schools, and other populated areas with varying intensity and with expanding range and precision. Initially, the mortars were capable of reaching distances of approximately 10 km/6 mi from the Gaza Strip, resulting in injuries, fatalities, and property damage in communities bordering Gaza. However, in more recent years, militant organizations have acquired rockets that can reach approximately 150 km/93 mi beyond Gaza [31]. During 2022–2023, most of rockets and mortar shells fired at Israel from the Gaza Strip targeted the communities surrounding the Gaza Strip and southern cities, but rockets were also fired at the metropolitan areas of Tel Aviv and Jerusalem [53], leaving a majority of the Israeli civilians (Jews and Arabs) in a state of ongoing insecurity.

In order to warn civilians of impending missile attacks, the Israeli government has installed a siren alarm system called "Red Color." Upon hearing an alarm, all civilians are required to enter the nearest protected area, preferably an in-house protected space or a bomb shelter. Pursuant to a law enacted in 1992, new buildings, homes and apartments must contain a protected space. This space, known as a "mamad," is built with reinforced concrete and air-tight steel enclosures for doors and windows. However, a large portion of the population in southern Israel, like the rest of the country, live in older buildings and do not have an in-home protected space. When there is an attack, such civilians use a shelter in their building, or an outside bomb shelter shared by residents of several buildings. When other options are not readily available, stairwells are considered better than other unprotected spaces.

The geographical proximity of a community to the Gaza Strip defines the time set between the siren and the attack. For example, the city of Ashkelon (population 153,000), located 10 km/6 mi from the Gaza Strip, receives a 30-s notice, whereas Sderot (population 33,000), which is only 3.7 km/2.3 mi away, receives a 15 s notice [34]. The time afforded is often insufficient to find shelter, especially for those without protected spaces in their residences, or those in the midst of their day-to-day activities outside the home.

For Jewish and Bedouin civilians in southern Israel, the threat of missile attacks is not a singular event, but an ongoing, unpredictable phenomenon. This harsh reality is intrinsic to life in southern Israel; there is no escape, and every individual is inevitably exposed. The situation disrupts daily life and creates an environment of chronic stress and trauma [17]. Thus, even after ceasefires are declared, many civilians continue their daily routine with a sense of caution and concern about the next missile attack [17, 44].

Studies have shown that continuous exposure to traumatic events, extreme violence, and risk to life can have a range of long-term mental health consequences including anxiety, depression, somatization, alcohol and drug abuse, and post-traumatic stress disorder (PTSD) [32, 45, 57]. It is estimated that 22.1% of people who have lived in settings affected by conflict in the preceding 10 years have a moderate or severe mental disorder at some point in time [15]. In a recent meta-analysis that focused on adult survivors of war between 1989 and 2019, the global point prevalence of PTSD was found to be 26.5% [24]. Somewhat surprisingly, prevalence rates were not significantly associated with war intensity and length, time elapsed since war, response rate, or survey quality [24].

The population of southern Israel consists of city and village-dwelling Jews and Bedouin Arabs.

The Bedouin population is an ethnic minority that differs from Jewish Israeli society by language, religion, and cultural characteristics [61]. Bedouins make up 3.5% of the Israeli population and 20% of Israel’s Southern district [21]. They possess a unique history and culture, and a distinct set of life-challenges [3]. Due to its high fertility rate, the Bedouin population is characterized by a high proportion of children (51.8% as compared to 32% among Jews and 33.8% among northern Arabs) [21].

Approximately 70% of the Bedouin population resides in permanent settlements (cities and organized villages recognized by the government, which provides municipal services), and approximately 30% live in unrecognized villages without formal municipal status and services such as electricity, water and sewer services, paved roads or educational facilities [56]. Traditionally a tribal, desert-dwelling nomadic society rooted in culturally and religiously conservative Muslim values, much of the Bedouin population has undergone substantial transformations including significant urbanization process [51, 60], increased participation in the job market [56], and a greater focus on formal education [61]. Nonetheless, Bedouin society remains deeply rooted in patriarchal and tribal structures. Even within urban areas, distinct tribes deliberately reside in separate neighborhoods in order to maintain separation from other tribes [3, 51]. While polygamy is prohibited by Israeli law, approximately one-third of Bedouin men still practice it [61], and consanguineous marriages remain common. Furthermore, many women are still restricted in their mobility, cannot travel without a male escort, and their social interactions are typically confined to the nuclear family [1]. Similarly, parental wishes and tribal boundaries limit women’s marriage options [3].

The literature suggested that the risk of PTSD varies among different racial and ethnic groups [16, 43, 46]. Although the mental health of the Jewish population in southern Israel following exposure to missile attacks has been studied, little is known about the psychological toll on adult Bedouin residents. A systematic review of 28 studies published from 2000 to 2016 that investigated the psychological impacts of continuous exposure to rocket fire on southern Israeli civilians revealed elevated levels of PTSD, depression, and other psychological reactions within the population [20]. These effects were noticeable during periods with limited missile attacks and were significantly higher during periods of escalated hostilities. Interestingly, none of the 28 studies investigated the impact of on-going incidents of missile attacks on the Bedouin population. Most of the studies comparing Jewish and Arab citizens focused on civilians residing in northern Israel. Several studies explored the psychological impact of missile attacks during the 2006 Second Lebanon War on Jewish and Arab children [46], adolescents [13] and adults [59] in northern Israel. Only two studies specifically focused on the Bedouins of southern Israel. Abu-Kaf et al. [2] compared coping resources and stress reactions among Jewish and Bedouin adolescents (aged 14–18 years) during a 2012 Israeli military operation in the Gaza Strip [2]. Other research has been conducted on the level of PTSD experienced among three-generation families (elderly parents, adult offspring, and adult grandchildren) of Jews, Arabs and Druze from both northern and southern regions of Israel following the 2006 Second Lebanon War and Operation Cast Lead in 2009 [43]. However, the number of southern Bedouin participants was not indicated in the study, and the author noted challenges in recruiting participants. Thus, there is a dearth of published research concerning differences in PTSD between the Jewish and Bedouin civilian populations in southern Israel. This study aims at achieving a better understanding of the unique experiences and needs of these communities regarding the mental health impacts of the on-going threat of missile attacks by investigating the prevalence of PTSD among both Jewish and Bedouin civilians living in southern Israel.

## Methods

### Study population

The study population included (a) adult Israeli civilians (n = 389) living in southern Israel, within 40 km/25 mi of the Gaza Strip (b) for at least 2 years. Participants above 18 years of age were recruited through Jewish and Bedouin social media. Readers of these posts were also asked to forward the study’s link to others (snowballing). While missiles from Gaza can now reach beyond southern Israel, the proximity criterion of 40 km/25 mi was chosen to capture data from individuals who were most subject to ongoing exposure [20].

### Procedure and ethical considerations

Data were collected via Hebrew and Arabic language self-administered questionnaires between January and March 2023 (before the ongoing war that started on October 7th, 2023). During the study period there were few missile attacks from Gaza, and no military operations by the Israeli Defense Force. Participants were informed that name identification was not required to participate; answering all questions was not required; and it was possible to freely withdraw from the study. No compensation or reward was given. The questionnaire was delivered through Google Forms. The introduction included an explanation of the study, its objectives, and a request to consent to participate. The Ethical Committee of the Nursing Department approved the study on January 15, 2023 (#4–23).

Strengthening the Reporting of Observational Studies in Epidemiology (STROBE) guidelines were used for reporting study results.

### Study variables

*Demographic characteristics*: Age (used as a continuous variable); Gender (male, female); Population group (Jewish, Bedouin); Marital status (married/living with a partner, single/divorced/widowed); Parental status (children, no children); Level of religiosity: secular [non-observant]; traditional [observes some religious practices] and religious/orthodox [observes most or all religious practices]; Education (school 1–12; professional; BA, MA or PhD); Self-reported socioeconomic status (SES) (not good, reasonable, good, excellent); Employment (unemployed, student, employed).

*Living Conditions*: Type of municipality (city, village); Type of residence (private house, apartment building, hut/tent); Existence of missile alarm system (yes, no); Existence of a protected space/shelter nearby (inside the living space, nearby, limited/no access); Length of residence in southern Israel (used as a continuous variable).

*Self-reported health* (bad, reasonable, good, very good, excellent); Existence of disability (motor, visual, auditory).

*PTSD symptoms* were assessed using the Hebrew and Arab version [40] of the Posttraumatic Stress Disorder Checklist (PCL-5); [11, 33]. The PCL-5 is a widely used DSM-correspondent self-report measure of PTSD symptoms [11] with a high level of internal reliability in prior studies [8, 29]. Participants responded to 20 items regarding how often they suffered from each PTSD symptom during the previous month. Items were rated on a 5-point Likert-type scale ranging from 0 (not at all) to 4 (extremely). Scores were calculated as the sum of the responses to all items, with higher scores indicating higher levels of PTSD symptoms. Importantly, there were no missing values for any of the subjects in any of the items. Study have used a score range of 33 as a cut-off point for PTSD [29, 33]. Previous studies have reported good reliability of the tool (Cronbach alpha = 0.95–0.96) [8, 29]. The questionnaire’s reliability by Cronbach’s alpha in this study was α = 0.98.

In addition, a probable PTSD diagnosis was made by assessing each item within the DSM-V clusters that are rated as 2 or higher as a qualifying score under that cluster [58]:

Questions 1–5 measure re-experiencing symptoms and at least one score of 2 and above is an indication of existence of this symptom.

Questions 6–7 measure avoidance symptoms and at least one score of 2 and above is an indication of existence of this symptom.

Questions 8–14 measure negative changes to cognition and mood and at least two scores of 2 and above is an indication of existence of this symptom.

Questions 15–20 measure hyper-arousal symptoms and at least two scores of 2 and above is an indication of existence of this symptom.

### Statistical analysis

The chi-square test was used to investigate associations between the Jewish and Bedouin participants, as well as demographic characteristics, living conditions, and health status in relationship to the presence of PTSD.

The association between the participants’ age and length of residence in southern Israel (continuous variables) and ethnic group was assessed using the Mann–Whitney Non-parametric Test, as was the association between these variables and the presence of PTSD.

Multivariable logistic regression with the Backward LR approach, with PTSD as a dependent variable, was used, while adjusting for variables found to be significantly associated with ethnic group (main independent variable) or PTSD, or both in univariate analyses. To avoid multicollinearity, Kendall's Tau coefficient was employed to examine the correlation between independent variables before their inclusion in the multivariable analysis. A high correlation was found between age and length of residence in southern Israel; age and education; level of religiosity and the population group; parental status and marital status; type of municipality and type of residence; self-reported physical condition and the existence of a disability, and the existence of in-house or nearby shelter and alarm system. Therefore, only one of the correlated variables was included in the multivariable regression. Consequently, the multivariable model included population group, age, marital status, self-reported SES, employment, settlement type, the existence of an alarm system, and disability. For all analyses, a value of *p* < 0.05 was considered statistically significant. Analyses were carried out with the SPSS version 25.0 statistical package (IBM, US).

## Results

### Demographic characteristics, living conditions and self-reported health status

The demographic characteristics of the study population are presented in Table 1. The study sample consisted of 389 Israeli civilians, 246 (63.2%) Jews and 143 (36.8%) Bedouins. Women accounted for 83% of the individuals in each group. The Jewish participants were significantly older than the Bedouins (mean age 36.6 (SD = 15.1) vs. 26.2 (SD = 9.5) years, *p* < 0.001). The proportion of married or living with a partner (69.9% vs. 37.1%), and parents of children (55.7% vs. 38.5%) was significantly higher among the Jewish participants in comparison to the Bedouins. Bedouin participants reported a significantly higher proportion of unemployment (28.7% vs. 10.7% among Jews), worse self-reported socioeconomic status (SES), and better self-reported health condition. No difference was observed between the Jews and Bedouins as to type of municipality (city vs. village). Among all participants, 50.6% lived in cities and 49.4% lived in villages. Importantly, among the Bedouins who lived in villages, nearly one-third (17% of the overall sample) lived in villages lacking government recognition as a municipal entity. In comparison to Jews, Bedouins reported a significantly higher proportion of inaccessibility to shelter (46.9% *vs*. 7.8%, *p* < 0.001), and lack of siren alert alarm system (25.2% vs. 0%, *p* < 0.001).

**Table 1:** Characteristics of the study population, Jews and Bedouins

Characteristics	Jews n = 246	Bedouins n = 143	*p-*value
Demographic characteristics			
*Gender* *n (%)*			
Male	42 (17.1)	24 (16.8)	NS
Female	204 (82.9)	119 (83.2)	
*Age, years*			
Mean (SD)	36.6 (15.1)	26.2 (9.5)	** < 0.001**
Median (IQR)	31.0 (25.0–44.0)	23.0 (20.0–29.0)	** < 0.001**
*Marital Status* *n (%)*			
Single/divorced/widowed	74 (30.1)	90 (62.9)	** < 0.001**
Living with a partner/married	172 (69.9)	53 (37.1)	
*Parental status* *n (%)*			
Children	137 (55.7)	55 (38.5)	**0.001**
No children	109 (44.3)	109 (44.3)	
*Level of religiosity* *n (%)*			
Secular	137 (55.7)	5 (3.5)	** < 0.001**
Traditional	61 (24.8)	12 (8.4)	
Religious/Orthodox	48 (19.5)	126 (88.1)	
*Education* *n (%)*			
School	70 (28.4)	57 (39.9)	**0.015**
Professional	42 (17.1)	13 (9.1)	
Academic, BA	102 (41.5)	65 (45.5)	
Academic, MA or PhD	32 (13.0)	8 (5.6)	
*Self-reported SES* *n (%)*			
Not good	15 (6.2)	11 (7.7)	**0.007**
Reasonable	75 (31.0)	65 (45.8)	
Good	125 (51.7)	48 (33.8)	
Excellent	27 (11.2)	18 (12.7)	
*Employment* *n (%)*			
Unemployed	26 (10.7)	41 (28.7)	** < 0.001**
Student	50 (20.5)	69 (48.3)	
Employed	168 (68.9)	33 (23.1)	
Living conditions			
*Type of municipality* *n (%)*			
City	118 (48.0)	79 (55.2)	NS
Village	128 (52.0)	64 (44.8)^†^	
*Type of residence* *n (%)*			
Private house	114 (60.5)	102 (71.3)	** < 0.001**
Apartment building	93 (39.1)	26 (18.2)	
Hut, tent	1 (0.4)	15 (10.5)	
*Existence of an alarm system*	100%	74.8	** < 0.001**
*Existence of a protected space (shelter) nearby* *n (%)*			
Inside the living space	205 (83.3)	62 (43.4)	** < 0.001**
Nearby	22 (8.9)	14 (9.8)	
No/limited access	19 (7.8)	67 (46.9)	
*Length of residence in southern Israel, years*			
Mean (SD)	23.0 (12.9)	13.3 (12.9)	** < 0.001**
Median (IQR)	23.0 (15.0–28.0)	12.0 (4.5–20.0)	** < 0.001**
Self-reported Health			
*Self-reported health condition* *n (%)*			
Bad	6 (2.4)	2 (1.4)	**0.005**
Reasonable	36 (14.6)	13 (9.1)	
Good	88 (35.8)	32 (22.4)	
Very Good	72 (29.3)	56 (39.2)	
Excellent	44 (17.9)	40 (17.9)	
*Existence of disability*	55 (22.3)	38 (26. 6)	**NS**

*NS* not significant, *SD* Standard deviation, *SES* socioeconomic status

Bold values indicate statistical significance

^†^Among the Bedouins who live in the villages, 32.8% (n = 24) live in unrecognized settlements

### PTSD symptoms by demographic and occupational characteristics (univariate analysis)

Among the respondents, 20.3% (41.3% Bedouins vs. 8.1% Jews, p < 0.001) surpassed the PCL-5 cutoff score of 33, indicative of PTSD. Differences in the frequency of PTSD were assessed by participants’ characteristics and are presented in Table 2. Single/divorced/widowed individuals had a significantly higher prevalence of PTSD than those married or living with the partner (31.7% vs. 12.0%). The reported prevalence of PTSD rose as the level of religiosity increased (7.0% among the secular, 15.1% among the traditional and 33.3% among the religious/orthodox). High self-reported SES and the status of being employed were associated with a significantly lower prevalence of PTSD. As to living conditions, prevalence of PTSD was higher among city dwellers vs. village residents, i.e., participants who lived in a hut/tent versus apartment building or house, and among participants who reported lack of accessibility to shelters and siren systems. PTSD prevalence was also higher among participants who reported having a disability. Among those with PTSD, length of residence in southern Israel was significantly shorter than those without PTSD (mean length of seniority = 15.3 years (SD = 9.3) vs. 20.8 years (SD = 13.3), p-value of t-test < 0.001. This data is not included in Table 2).

**Table 2:** Frequency of PTSD by demographic characteristics, living conditions, and health status

Characteristics	PTSD (%)	*p*-value
*Ethnic Group*		
Jews	8.1	
Bedouins	41.3	** < 0.001**
*Gender*		
Male	22.7	
Female	19.8	0.052
*Marital status*		
Single/divorced/widowed	31.7	
Living with a partner/married	12.0	** < 0.001**
*Parental status*		
Children	16.1	
No children	24.4	0.058
*Level of religiosity*		
Secular	7.0	
Traditional	15.1	** < 0.001**
Religious/Orthodox	33.3	
*Education*		
School	24.4	
Professional	14.5	0.276
Academic (BA, MA or PhD)	19.3	
*Self-reported SES*		
Not good	50.0	
Reasonable	28.6	** < 0.001**
Good	11.0	
Excellent	13.3	
*Employment*		
Unemployed	31.3	
Student	27.7	** < 0.001**
Employed	12.4	
*Type of municipality*		
City	25.9	
Village	14.6	**0.008**
*Type of residence*		
Private house	17.9	
Apartment building	21.8	**0.008**
Hut, tent	50.0	
*Existence of an alarm system*		
Yes	17.3	
No	48.6	** < 0.001**
*Existence of a protected space (shelter) nearby*		
Inside the living space	14.6	
Nearby	27.8	** < 0.001**
No/limited approach	34.9	
*Self-reported health*		
Bad	25.0	
Reasonable	26.5	0.8
Good	20.0	
Very Good	19.5	
Excellent	17.9	
*Existence of disability*		
Yes	32.3	
No	16.6	**0.002**

*PTSD* post-traumatic stress disorder, *SES* socioeconomic status

Bold values indicate statistical significance

**Fig. 1:**
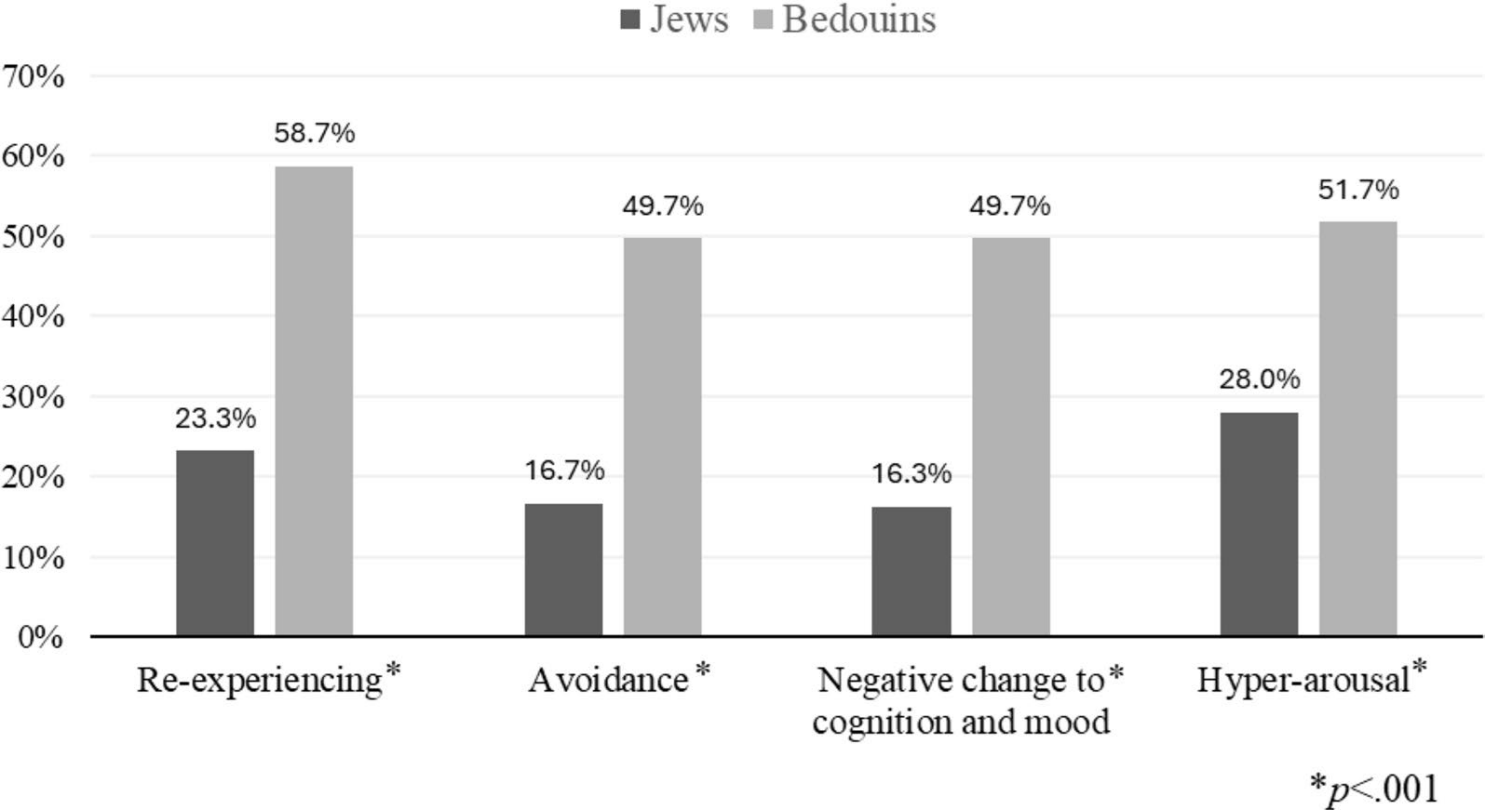
PCL-5 subscales among Jewish and Bedouin respondents

Frequencies of experiencing one or more symptoms ranged from 16.3 to 58.7% across the four PCL-5 symptom clusters, with significantly higher scores among Bedouins compared to Jews: twice as high in re-experiencing and hyper-arousal and three times higher in avoidance and negative changes to cognition and mood (p < 0.001 for all subscales). (Fig. 1).

When the total PCL-5 score and each subscale were analyzed as continuous variables (Table 3), the differences between Jews and Bedouins in the total PCL-5 score and in each subscale were statistically significant. Bedouins had higher median scores in the Re-experiencing, Negative Changes to Cognition and Mood, and Hyperarousal subscales (p < 0.001, Mann–Whitney non-parametric test).

**Table 3:** PCL-5 total and subscales by ethnic group

PCL-5 total and subscales	Jews		Bedouins		Total	
	Mean (SD)	Median (IQR)	Mean (SD)	Median (IQR)	Mean (SD)	Median (IQR)
Re-experiencing subscale*	0.6 (1.2)	0 (0–0)	2.0 (2.1)	1.0 (0–5)	1.1 (1.8)	0 (0–2)
Avoidance subscale*	0.3 (0.6)	0 (0–0)	0.9 (0.9)	0 (0–2)	0.5 (0.8)	0 (0–1)
Negative changes to cognition and mood*	0.7 (1.5)	0 (0–1)	2.8 (3.0)	1 (0–7)	1.5 (2.4)	0 (0–2)
Hyperarousal*	1.1 (1.7)	0 (0–2)	2.5 (2.6)	2 (0–6)	1.6 (2.2)	0 (0–3)
PCL-5 total*	10.0 (14.2)	5 (0–13)	26.8 (22.7)	22 (6–40)	16.2 (19.5)	8.0 (1–24)

*SD* Standard Deviation, *IQR* Interquartile range

*p of Mann–Whitney Test non-parametric test for the difference between Jews and Bedouins < .001

### Odds for PTSD (multivariable analysis)

The influence of interaction between the population group (Jews/Bedouins) and all independent variables on odds for PTSD was tested, and all interactions were not statistically significant or even borderline significant.

Multivariable analysis (Table 4) revealed that after adjustment for demographic and household characteristics, Bedouin participants had almost six-fold significant odds of having PTSD (OR 5.6, 95%CI 2.8–10.8) compared to Jewish participants. Individuals who were single vs. married or living with the partner, and those living in urban areas (vs. rural) exhibited more than two-fold significant odds of having PTSD. Additionally, participants with disability had almost three-fold significant odds of PTSD in comparison to those who did not report disability (OR 2.7, 95%CI 1.4–5.1). Participants with self-reported low socioeconomic status (SES) had a six-fold significant odds compared to those with good or excellent SES (OR 6.0, 95%CI 2.2–16.5). Participants who did not have an alarm system had more than two-fold odds for PTSD (OR 2.3, 95%CI 1.1–5.5). The final model explained 37% of the variance in PTSD. After adjustment for demographic characteristics, living conditions, and age.

**Table 4:** Odds for PTSD, demographic characteristics, living conditions, and health status

Characteristics	n	PTSD n (%)	Odds for PTSD	
	389	79 (20.3%)	Odds Ratio (OR)	95% CI
*Population group*				
Jews	246	20 (8.1)	Ref	–
Bedouins	143	59 (41.3)	**5.6**	**2.8–10.8**
*Family status*				
Single/divorced/widowed	164	53 (31.7)	**2.3**	**1.2–4.2**
Living with a partner/married	225	27 (12.0)	Ref	–
*Self-reported SES*				
Not good	26	13 (50.0)	**6.0**	**2.2–16.5**
Reasonable	140	40 (28.6)	**2.3**	**1.2–4.2**
Good/Excellent	218	25 (11.5)	Ref	–
*Type of municipality*				
City	197	51 (25.9)	**2.1**	**1.1–3.8**
Village	192	28 (14.6)	Ref	–
*The existence of an alarm system*				
Yes	352	61 (17.3)	Ref	–
No	37	18 (48.6)	**2.3**	**1.1–5.5**
*Existence of disability*				
Yes	93	30 (32.3)	**2.7**	**1.4–5.1**
No	296	49 (16.6)	Ref	**–**

*PTSD* post-traumatic stress disorder, *Ref* reference, *SES* socioeconomic status

Bold values indicate statistical significance

The model included all variables mentioned in the Table and age

The final model explains 37% of the variance in PTSD

## Discussion

Our study aimed to assess the prevalence of PTSD among civilians living in southern Israel. To our knowledge, this is the first study to compare the mental health state of the adult Jewish and Bedouin sub-populations in the face of past missile attacks and the ongoing threat of future missile attacks.

Overall, 20.3% of the respondents exhibited PTSD symptoms during a period of relative calm. Israel’s Operation Breaking Dawn, during which 11,175 were launched at southern Israel from Gaza, injuring 70 Israelis and damaging homes and factories, occurred August 5–7, 2022, almost 6 months before the study period [52]. These findings align with the results of the Green et al. [20] systematic review of studies conducted in Israel, which reported PTSD rates ranging from 5.6 to 35.2% during periods of relative calm [20]. In the present sample, 8.1% of the Jewish participants reported symptoms characteristic of PTSD during the previous month. A study conducted using a sample of 764 Jewish residents of central and southern Israel, 4 months after Israel’s 2014 Operation Protective Edge, revealed similar results, in that 8.2% of the participants exhibited symptoms of PTSD [8]. Studies conducted during times of active military conflict consistently found higher prevalence rates [9, 35]. For example, a cross-sectional study among Jewish students from a college near Sderot during active conflict reported a PTSD rate of 20% [9]. While comparing the prevalence of PTSD in this and other studies, it’s important to take into account, that this prevalence is influenced by the proportion of women in the study population. Previous studies have shown that females are more likely to participate in online surveys [50]. Thus, in our study population females comprise 80%. Women are at a higher risk of developing PTSD compared to men, even when facing similar types of trauma. Several explanations have been proposed for this gender disparity. For instance, it has been suggested that women might be more vulnerable to mental health consequences due to experiencing trauma within established relationships or enduring chronic traumatic exposures, such as ongoing interpersonal violence within marriage. Another hypothesis posits that women's gendered social roles, like being a wife, mother, or caretaker, could exacerbate the negative effects of trauma exposure. This is because women may face additional strain when traumatic experiences or stress reactions impede their ability to fulfill these roles [26, 39, 54]. On the other hand, the predominance of females in our study is not only an issue specific to this research but also present in previous ones. In the study of 251 Jewish Israeli adults living in communities, exposed to rocket fire, 19.5% of the study population suffered from severe PTSD, while 80% of the sample were women [10]. Most participants (80%) were women also in the study on 501 residents living within the range of missile attack [19] and 75% were women among the participants from Sderot [38]. Thus, the comparison of the findings in our study is valid, as well as a comparison between Bedouins and Jews in the current study, as the proportion of women in the sample of Jews and Bedouins was similar.

The present study revealed that the odds for PTSD were significantly higher—by almost six-fold—for Bedouins compared to Jews, after taking into account possible confounders. This finding is in line with previous Israeli studies which have consistently shown that Israeli Arabs exhibit higher levels of PTSD following traumatic events compared to their Jewish counterparts [23, 43, 59]. The grades were higher for Bedouins in every subscale.

There are several plausible explanations for the increased prevalence of PTSD within the Bedouin population. The Jewish population has experienced several wars and periods of domestic terrorism. For most Israeli Jews, mandatory military service for men and women is an accepted part of life. In contrast, it was not until after 2005 that Bedouin communities in southern Israel began to find themselves subject to life-threatening missile attacks. Most Bedouin citizens, for whom Israeli Army service is optional, have not been deliberately targeted by Israel’s enemies during wars or periods of domestic terrorism, and thus have had less exposure to them and less experience in coping with them. Only 300–500 Bedouins per year decide to enlist in the army, and only one-third of the enlistees are residents of southern Israel [55]. Most Bedouin enlistees come from northern Israel, where they are more integrated into Israeli society than are Bedouins in southern Israel [55]. Reportedly, Bedouins from southern Israel who enlist in the army face criticism and even threats of violence from their communities [55].

Studies suggest increased prevalence of PTSD among Bedouins may be related to the “conflict of belonging” [43, 44, 59]. An Israeli study about the level of perceived distress in university medical students during a prolonged period of terror attacks reported an association between greater sense of national belonging and lower level of distress [28]. The close familial connections between Bedouin residents of Gaza and Bedouin residents in southern Israel may render the latter susceptible to secondary traumatization. Furthermore, although Bedouins are Israeli citizens, a growing number of Bedouins identify themselves as Palestinians [43], leading to the stressful dilemma of dual allegiance on both familial and community levels. Another possible contributing factor is that some Israeli Bedouins report feelings of discrimination [3], which may increase the likelihood of adverse mental health reactions [14].

By history and culture, Bedouins feel bound to their living places and their tribes. In contrast, Jewish citizens feel they have alternative housing options throughout Israel. While remaining in southern Israel is a choice for Jewish citizens, Bedouins do not feel free to leave their living places or violate tribal obligations [3]. We believe, that much lower prevalence of PTSD among Jews may result from several factors. One of them is the strong sense of security and social support in Jewish communities, which can reduce exposure to trauma and associated stressors [22]. Additionally, a higher proportion of Jews have in-house shelters and missile alarm systems, which may reduce the sense of fear at the time of attack. Fear has been documented as playing a key role in posttraumatic symptoms. A subjective sense of fear was found to be even more strongly related to posttraumatic symptoms than the actual objective exposure [47]. Another contributing factor could be their adaptation to the situation, as residents in these areas have often experienced repeated exposure to rocket attacks over time. Prolonged periods of threat exposure strengthen individuals' psychological resources, enabling them to cope better with potential missile attacks, compared to populations experiencing these events for the first time [12].

A significant difference between unemployment rate and SES among Bedouin and Jewish participants raises the possibility that these factors must be addressed to a greater extent than other factors that may be impacted by policy makers. Undeniably, the Bedouin population in Israel is one of the most economically disadvantaged groups [61], which may be attributed to a combination of a high fertility rate and higher unemployment. The shift in Bedouin economic activity from animal husbandry to wage labor places Bedouin workers in stressful competition for non-professional jobs [56]. The unemployment rate is especially high among Bedouin women. According to Knesset Israel [27], in 2019, 77% of Bedouin women of prime employable age were not part of the workforce, compared with 62% among Arab women in general, and 14% among Jewish women. Those Bedouin women who are employed earn, on average, 45% less than their Jewish counterparts [27]. One of the main factors behind the relatively low employment rates for Arab men and women is low education level. With regard to higher education, the rate of eligibility for a matriculation certificate among Bedouin high school students is significantly lower compared to Arabs in general, and compared to the Jewish population (48.1%, 63.9%, and 73.1% respectively) [21].

This study used self-reported SES, which was found significantly associated with increased odds for PTSD with a dose–response relationship independent of other participant characteristics. Individuals in low socio-economic positions may experience a higher prevalence of adverse events, such as poverty, discrimination, violence, and crime. These chronic stressors can contribute to the development of PTSD, as they expose individuals to traumatic situations more frequently [37]. Studies reveal that individuals with lower SES often face barriers in accessing healthcare and mental health services [25, 30, 62]. This barriers can impede ability to receive timely and appropriate treatment for traumatic experiences, thereby elevating the risk of developing PTSD [20, 30]. Apart from the influence of low SES, and limited access to overall healthcare, there appears to be under-utilization of mental health services among minority groups [36, 42]. Thus, studies indicate low utilization of mental health services among Bedouin citizens of Israel, which can be attributed to language barriers, mistrust in the quality of treatments, fear of psychological intervention, and concerns about social stigma associated with mental illness [5, 18, 48]. Moreover, within Arab and Bedouin culture, there is a prevailing belief that problems should be resolved within the family rather than through outside professional assistance [6, 18].

Certain household characteristics can play a role in influencing anxiety level during exposure to a missile attack, including the availability of a shelter and the presence of an alarm system. In comparison to Jews, Bedouin citizens had limited access to shelters and siren systems. This discrepancy may be attributable to the fact that 17% of the Bedouins in the sample resided in unrecognized villages lacking municipal infrastructure and services. Before Bedouin villages were subject to missile attacks, there was no need for shelters and alarm systems. When the possibility of a missile attack increased, the need for such infrastructure increased. The lack of accessibility to shelter was significantly associated with PTSD, and lack of siren system was significantly associated with PTSD in the multivariable analysis.

Our study revealed that citizens residing in urban areas vs. rural areas, had a two-fold significant likelihood of developing PTSD. This observation also aligns with the results of the systematic review cited above [20]. The potential explanation for this phenomenon may lie in the distinctive nature of small Israeli villages and communities. These “kibbutzim” and “moshavim” were initially established to farm and settle the land, driven by ideals of economic and social equality, mutual support, and individual responsibility. Nuttman-Shwartz et al. [38] proposed that the sense of belonging to such communities serves as a valuable resource, strong enough to reduce posttraumatic distress and increase resilience [38].

Finally, having a disability was associated with almost three-fold higher odds of PTSD, after considering other characteristics. Shpigelman and Gelkopf [49] investigated the experiences and obstacles faced by Israelis with lifelong disabilities during periods of conflict escalation [49]. The findings revealed that participants with physical and sensory disabilities encountered challenges in reaching shelters within the limited time-frame between the sounding of the siren and an actual attack, and challenges in obtaining adequate safety information. Additionally, increased reliance on others, and the perceived overprotectiveness imposed by family members during war, contributed to the distress and anxiety of disabled persons during emergency situations [49].

Importantly, the research was conducted during a peaceful interval, free from conflict. However, the outbreak of war on October 7, 2023, had widespread ramifications, impacting various sectors, including the Bedouin community. Civilian Bedouins endured injuries, fatalities, and even abductions. The findings of this study underscore the imperative to allocate resources and focus on aiding this population in coping with the mental challenges they face.

## Limitations

The gold standard for diagnosing PTSD is a structured clinical interview. While the PCL-5 questionnaire is a reliable self-report measure for DSM-5 PTSD symptoms and is useful for screening, monitoring symptom severity, and establishing a provisional diagnosis, it cannot be used to formally diagnose PTSD [11, 33].

In our study, the possibility of an ascertainment bias stemming from the recruitment method, which solely relies on social media, cannot be excluded. Although the recent study found no internet accessibility barriers within Arab population in Israel [41] previous study, conducted during the Covid-19 pandemic reported such barriers in the Arab population [7]. We believe that internet infrastructure and internet literacy in both recognized and unrecognized Bedouins’ villages in the Negev may influence the representativity of the sample toward younger and more educated individuals, having access to the internet. Another limitation may be related to snowball sampling methods for participant recruitment. This approach, while practical for reaching a large and diverse population, can introduce biases. It is important to note that this method was consistently applied across both the Bedouin and Jewish populations. Consequently, any potential biases introduced by the sampling method would similarly affect both groups, rather than disproportionately influencing one group over the other. Despite this uniform application, the snowball sampling method can still lead to the overrepresentation of certain subgroups within the initial respondents, potentially magnifying specific characteristics, such as higher PTSD rates. To mitigate this, we ensured that our initial sample included individuals from various demographics and backgrounds in both populations. Furthermore, we conducted preliminary analyses to check for significant differences in PTSD rates among early and later respondents within each group. Our findings showed no significant differences, suggesting that the initial PTSD rates were not disproportionately amplified within either population. Additionally, we applied statistical adjustments to account for potential biases introduced by the snowball sampling method.

We acknowledge that these measures cannot entirely eliminate the limitations inherent in the sampling method. Therefore, our findings should be interpreted with caution, considering these potential biases. Future research should aim to replicate our results using random sampling methods to further validate our conclusions and minimize the impact of any sampling-related biases.

Given the cross-sectional design of the study, the authors cannot draw conclusions about the causality of the associations found in the study. Another limitation is that we did not collect data about pre-existing mental health and exposure to traumatic events other than missile attacks. Therefore, the increased prevalence of PTSD among Bedouins may be related to higher exposure rates to other traumatic events. According to a 2019 study encompassing the entire Israeli Arab society, 75% of respondents from the Negev reported a lack of personal security due to violence in their community. Additionally, the percentage of Negev respondents who indicated they had been affected by violence of any kind (excluding sexual violence) was the highest among all groups within Arab society [4].

Further research should examine previous mental health history and previous exposure to other traumatic events and violence.

Although self-reported SES was taken into account in the multivariable model, it is possible that its influence was not sufficiently separated out.

## Policy implications and recommendations

Healthcare professionals must proactively address the mental impacts of missile attacks on all civilians. This includes screening for the condition and providing tailored treatment and support, considering individuals’ language, cultural background, and other characteristics that may be associated with PTSD. To enhance service acceptance, healthcare providers and authorities should seek to eliminate barriers, including geographical distance, inadequate availability of mental health services, especially in unrecognized villages, cost, stigma, and mistrust. This may be aided by avoiding psychopathology labels, providing free-of-charge treatment, and ensuring easy accessibility by delivering services within Bedouin communities. Authorities should address the disparity in bomb shelter access and siren warning coverage between Bedouin and Jewish communities.

Our findings are relevant to other war-stricken areas around the world. Conducting thorough mental health assessments, along with evaluating the awareness of available mental health services, is of utmost importance, particularly within disadvantaged populations in communities subject to missile attack or other elements of warfare. Moreover, establishing easily accessible, community-based, culturally competent mental health services that are affordable or free-of-charge is crucial for effectively addressing the specific needs of disadvantaged individuals. Effective programs must employ practitioners who actively engage within the community beyond the clinic setting. Representatives from disadvantaged communities should be involved from the planning and implementation of the interventions to the evaluation of their efficacy. It is also essential to employ ethnic minority practitioners to enhance recruitment, retention in care, and recovery efforts.

## Conclusions

The findings of this study demonstrate a six times higher prevalence of PTSD among the Bedouin population than among Jews, highlighting the necessity for culturally sensitive mental health interventions tailored to this community. Such interventions are essential not only to address current PTSD symptoms but also to prepare them for possible similar situations in the future. Several sociodemographic characteristics that significantly increase the probability of having PTSD were detected, the most prominent of which was low SES.

## Data Availability

The analyzed dataset and the syntax file are available from the corresponding author upon reasonable request. Part of the dataset is in Hebrew.

## References

[1] Abu Rabia R, Hendel T, Kagan I. Views of Bedouin physicians and nurses on nursing as a profession in Israel: there is more to strive for. Nurs Health Sci. 2021;23(2):498–505. 10.1111/nhs.12834. 33793072 10.1111/nhs.12834

[2] Abu-Kaf S, Braun-Lewensohn O, Kalagy T. Youth in the midst of escalated political violence: sense of coherence and hope among Jewish and Bedouin Arab adolescents. Child Adolesc Psychiatry Ment Health. 2017;11(1):42. 10.1186/s13034-017-0178-z. 28855964 10.1186/s13034-017-0178-z PMC5574240

[3] Alhuzail NA, Segev E. The challenges of Young Bedouin men living in a changing society. Am J Orthopsychiatry. 2023;93(1):97–106. 10.1037/ort0000658. 36634010 10.1037/ort0000658

[4] Ali N, Lewin-Chen R, Najami-Yousef O. Violence, crime and policing in Arab society: personal and community security index 2019. 2020. https://abrahaminitiatives.org.il/2020/06/29/%D7%90%D7%9C%D7%99%D7%9E%D7%95%D7%AA-%D7%A4%D7%A9%D7%99%D7%A2%D7%94-%D7%95%D7%A9%D7%99%D7%98%D7%95%D7%A8-%D7%91%D7%99%D7%99%D7%A9%D7%95%D7%91%D7%99%D7%9D-%D7%94%D7%A2%D7%A8%D7%91%D7%99%D7%99%D7%9D/

[5] Al-Krenawi A, Graham JR. A comparative study of family functioning, health, and mental health awareness and utilization among female Bedouin-Arabs from recognized and unrecognized villages in the Negev. Health Care Women Int. 2006;27(2):182–96. 10.1080/07399330500457978. 16484161 10.1080/07399330500457978

[6] Al-Krenawi A, Graham JR. Mental health help-seeking among Arab university students in Israel, differentiated by religion. Ment Health Relig Cult. 2011;14(2):157–67. 10.1080/13674670903454229. 10.1080/13674670903454229

[7] Axelrad H, Matar H, Tehawkho M. Challenge Facing Israeli Arab Society The Digital. 2021. https://www.idc.ac.il/he/research/aiep/pages/policy-papers.aspx.

[8] Ben-Tzur N, Zanbar L, Kaniasty K. Mastery, social support, and sense of community as protective resources against psychological distress among israelis exposed to prolonged rocket attacks. J Trauma Stress. 2021;34(3):501–11. 10.1002/jts.22629. 33219713 10.1002/jts.22629

[9] Besser A, Neria Y. When home isn’t a safe haven: insecure attachment orientations, perceived social support, and PTSD symptoms among Israeli evacuees under missile threat. Psychol Trauma Theory Res Pract Policy. 2012;4(1):34–46. 10.1037/a0017835. 10.1037/a0017835

[10] Besser A, Zeigler-Hill V, Weinberg M, Pincus AL, Neria Y. Intrapersonal resilience moderates the association between exposure-severity and PTSD symptoms among Civilians exposed to the 2014 Israel-Gaza conflict. Self Identity. 2015. 10.1080/15298868.2014.966143. 10.1080/15298868.2014.966143

[11] Blevins CA, Weathers FW, Davis MT, Witte TK, Domino JL. The posttraumatic stress disorder checklist for DSM-5 (PCL-5): development and initial psychometric evaluation. J Trauma Stress. 2015;28:489–98. 10.1002/jts. 26606250 10.1002/jts

[12] Braun-Lewensohn O, Mosseri Rubin M. Personal and communal resilience in communities exposed to missile attacks: does intensity of exposure matter? J Positive Psychol. 2014. 10.1080/17439760.2013.873946. 10.1080/17439760.2013.873946

[13] Braun-Lewensohn O, Sagy S, Roth G. Coping strategies among adolescents: Israeli Jews and Arabs facing missile attacks. Anxiety Stress Coping. 2009;23(1):35–51. 10.1080/10615800802647601. 10.1080/10615800802647601 19326275

[14] Brooks Holliday S, Dubowitz T, Haas A, Ghosh-Dastidar B, DeSantis A, Troxel WM. The association between discrimination and PTSD in African Americans: exploring the role of gender. Ethn Health. 2020;25(5):717. 10.1080/13557858.2018.1444150. 29490467 10.1080/13557858.2018.1444150 PMC6113108

[15] Charlson F, van Ommeren M, Flaxman A, Cornett J, Whiteford H, Saxena S. New WHO prevalence estimates of mental disorders in conflict settings: a systematic review and meta-analysis. The Lancet. 2019;394(10194):240–8. 10.1016/S0140-6736(19)30934-1. 10.1016/S0140-6736(19)30934-1 PMC6657025 31200992

[16] Cruz-Gonzalez M, Alegría M, Palmieri PA, Spain DA, Barlow MR, Shieh L, Williams M, Srirangam P, Carlson EB. Racial/ethnic differences in acute and longer-term posttraumatic symptoms following traumatic injury or illness. Psychol Med. 2022. 10.1017/s0033291722002112. 35903010 10.1017/s0033291722002112 PMC9884321

[17] Diamond GM, Lipsitz JD, Fajerman Z, Rozenblat O. Ongoing traumatic stress response (OTSR) in Sderot, Israel. Prof Psychol Res Pract. 2010;41(1):19–25. 10.1037/A0017098. 10.1037/A0017098

[18] Fahoum K, Al-Krenawi A. Perceptions of stigma toward mental illness in Arab society in Israel. Int Soc Work. 2023;66(3):769–80. 10.1177/00208728211018727/FORMAT/EPUB. 10.1177/00208728211018727/FORMAT/EPUB

[19] Gil S, Weinberg M, Or-Chen K, Harel H. Risk factors for DSM 5 PTSD symptoms in Israeli civilians during the Gaza war. Brain Behav. 2015;5(4):1–9. 10.1002/BRB3.316. 25905028 10.1002/BRB3.316 PMC4402039

[20] Greene T, Itzhaky L, Bronstein I, Solomon Z. Psychopathology, risk, and resilience under exposure to continuous traumatic stress: a systematic review of studies among adults living in Southern Israel. Traumatology. 2018;24(2):83–103. 10.1037/TRM0000136. 10.1037/TRM0000136

[21] Haj-Yahya N, Khalaily M, Rudnitzky A, Fargeon B. Statistical Report on Arab Society in Israel. 2022.

[22] Henrich CC, Shahar G. Social support buffers the effects of terrorism on adolescent depression: findings from Sderot, Israel. J Am Acad Child Adolesc Psychiatry. 2008;47(9):1073–6. 10.1097/CHI.0B013E31817EED08. 18664998 10.1097/CHI.0B013E31817EED08

[23] Hobfoll SE, Canetti-Nisim D, Johnson RJ, Palmieri PA, Varley JD, Galea S. The association of exposure, risk, and resiliency factors with PTSD among Jews and Arabs exposed to repeated acts of terrorism in Israel. J Traumat Stress. 2008;21(1):9–21. 10.1002/jts.20307. 10.1002/jts.20307 PMC2802182 18302179

[24] Hoppen TH, Priebe S, Vetter I, Morina N. Global burden of post-traumatic stress disorder and major depression in countries affected by war between 1989 and 2019: a systematic review and meta-analysis. BMJ Glob Health. 2021. 10.1136/bmjgh-2021-006303. 34321235 10.1136/bmjgh-2021-006303 PMC8319986

[25] Kim MK, Lee SM, Bae SH, Kim HJ, Lim NG, Yoon SJ, Lee JY, Jo MW. Socioeconomic status can affect pregnancy outcomes and complications, even with a universal healthcare system. Int J Equity Health. 2018;17(1):2. 10.1186/s12939-017-0715-7. 29304810 10.1186/s12939-017-0715-7 PMC5756361

[26] Kimerling R, Weitlauf JC, Iverson KM, Karpenko JA, Jain S. Gender Issues in PTSD. In: Handbook of PTSD: Science and Practice. 2014.

[27] Knesset Israel. Bedouin women earn 45% less than their Jewish counterparts. Knesset News. 2022. https://main.knesset.gov.il/en/news/pressreleases/pages/press1322q.aspx

[28] Kovatz S, Kutz I, Rubin G, Dekel R, Shenkman L. Comparing the distress of American and Israeli medical students studying in Israel during a period of terror. Med Educ. 2006;40(4):389–93. 10.1111/j.1365-2929.2006.02409.x. 16573676 10.1111/j.1365-2929.2006.02409.x

[29] Levi-Belz Y, Yalon S. Depression, PTSD, and suicidal ideation among ex-ultra-Orthodox individuals in Israel. Eur J Psychotraumatol. 2023. 10.1080/20008066.2023.2172259. 37052115 10.1080/20008066.2023.2172259 PMC9930855

[30] McMaughan DJ, Oloruntoba O, Smith ML. Socioeconomic status and access to healthcare: interrelated drivers for healthy aging. Front Public Health. 2020;8:231. 10.3389/fpubh.2020.00231. 32626678 10.3389/fpubh.2020.00231 PMC7314918

[31] Ministry of Foreign Affairs. (n.d.). *Range of Fire from Gaza*. Retrieved June 5, 2023, from https://embassies.gov.il/MFA/AboutIsrael/Maps/Pages/Range-of-Fire-from-Gaza.aspx

[32] Murthy RS, Lakshminarayana R. Mental health consequences of war: a brief review of research findings. World Psychiatry. 2006;5(1):25–30. 16757987 PMC1472271

[33] National Center for PTSD. (2018). Using the PTSD Checklist for DSM-5 (PCL-5) www.ptsd.va.gov (Vol. 5).

[34] National Emergency. (n.d.). National Emergency Portal: Getting to a Shelter. Retrieved May 13, 2023, from https://www.oref.org.il/12487-17292-en/Pakar.aspx

[35] Neria Y, Besser A, Kiper D, Westphal M. A longitudinal study of posttraumatic stress disorder, depression, and generalized anxiety disorder in Israeli civilians exposed to war trauma. J Trauma Stress. 2010;23(3):322–30. 10.1002/JTS.20522. 20564364 10.1002/JTS.20522

[36] Norris FH, Alegria M. Mental health care for ethnic minority individuals and communities in the aftermath of disasters and mass violence. CNS Spectr. 2005;10(2):132–40. 10.1017/S1092852900019477. 15685124 10.1017/S1092852900019477

[37] Nurius PS, Uehara E, Zatzick DF. Intersection of stress, social disadvantage, and life course processes: reframing trauma and mental health. Am J Psychiatr Rehabil. 2013;16(2):91–114. 10.1080/15487768.2013.789688. 25729337 10.1080/15487768.2013.789688 PMC4343539

[38] Nuttman-Shwartz O, Dekel R, Regev I. Continuous exposure to life threats among different age groups in different types of communities. Psychol Trauma Theory Res Pract Policy. 2015;7(3):269–76. 10.1037/a0038772. 10.1037/a0038772 25793400

[39] Olff M, Langeland W, Draijer N, Gersons BPR. Gender differences in posttraumatic stress disorder. Psychol Bull. 2007;133(2):183–204. 10.1037/0033-2909.133.2.183. 17338596 10.1037/0033-2909.133.2.183

[40] Palgi S, editor. A collection of self-report questionnaires. Jerusalem: Clinical Psychology Professional Committee, Ministry of Health; 2019.

[41] Penn N, Laron M. Use and barriers to the use of telehealth services in the Arab population in Israel: a cross sectional survey. Isr J Health Policy Res. 2023. 10.1186/s13584-023-00569-6. 37221598 10.1186/s13584-023-00569-6 PMC10204005

[42] Roberts AL, Gilman SE, Breslau J, Breslau N, Koenen KC. Race/ethnic differences in exposure to traumatic events, development of post-traumatic stress disorder, and treatment-seeking for post-traumatic stress disorder in the United States. Psychol Med. 2011;41(1):71–83. 10.1017/S0033291710000401. 20346193 10.1017/S0033291710000401 PMC3097040

[43] Ron P. Posttraumatic stress disorder among three-generation families in times of war: a comparison between Israeli Jewish and Arabs after the second Lebanon war (2006) and cast lead operation (2009). Traumatology. 2014;20(4):269–76. 10.1037/h0099863. 10.1037/h0099863

[44] Ron P. PTSD, ASD, secondary-traumatization, and death-anxiety among civilians and professionals as outcomes of on-going wars, terror attacks and military operations: an integrative view. Psychology. 2019;10(12):1688–710. 10.4236/psych.2019.1012111. 10.4236/psych.2019.1012111

[45] Rozanov V, Franciškovic T, Marinic I, Macarenco MM, Letica-Crepulja M, Mužinic L, Jayatunge R, Sisask M, Vevera J, Wiederhold B, Wiederhold M, Miller I, Pagkalos G. Mental health consequences of war conflicts. Adv Psychiatry. 2018. 10.1007/978-3-319-70554-5_17/COVER. 10.1007/978-3-319-70554-5_17/COVER

[46] Sandler L, Sommerfeld E, Shoval G, Tsafrir S, Chemny A, Laor N, Zalsman G. Effects of ethnicity on sub-clinical PTSD and depressive symptoms, following exposure to missile attacks in Israel: a pilot study. Int J Psychiatry Clin Pract. 2015;19(1):51–5. 10.3109/13651501.2014.980829. 25356662 10.3109/13651501.2014.980829

[47] Shechory-Bitton M. The impact of repetitive and chronic exposure to terror attacks on israeli mothers’ and children’s functioning. Isr J Psychiatry Relat Sci. 2013;50(3):157–63. 24622474

[48] Shorer S, Caspi Y, Goldblatt H, Azaiza F. Acknowledging post-traumatic stress disorder: treatment utilisation amongst Israeli Bedouin and Jewish combat veterans. Br J Soc Work. 2021;51(2):389–407. 10.1093/bjsw/bcaa152. 10.1093/bjsw/bcaa152

[49] Shpigelman CN, Gelkopf M. The experiences and needs of individuals with disabilities exposed to chronic political violence. Disabil Rehabil. 2017;39(1):23–35. 10.3109/09638288.2016.1138557. 26879275 10.3109/09638288.2016.1138557

[50] Smith WG. Does gender influence online survey participation? San Jose: San Jose State University; 2008.

[51] Tamari S, Katoshevski R, Karplus Y, Dinero SC. Urban tribalism: negotiating form, function and social milieu in Bedouin towns, Israel. City Territ Archit. 2016. 10.1186/s40410-016-0031-3. 10.1186/s40410-016-0031-3

[52] The Meir Amit Intelligence and Terrorism Information Center. (2022). Escalation in the Gaza Strip - Summary of Operation Breaking Dawn (August 5–7, 2022).

[53] The Meir Amit Intelligence and Terrorism Information Center. (2023). Operation “Shield and Arrow” In the Gaza Strip - Summary. https://www.terrorism-info.org.il/en/operation-shield-and-arrow-in-the-gaza-strip-summary/

[54] Tolin DF, Foa EB. Sex differences in trauma and posttraumatic stress disorder: a quantitative review of 25 years of research. Psychol Bull. 2006;132(6):959–92. 10.1037/0033-2909.132.6.959. 17073529 10.1037/0033-2909.132.6.959

[55] Toth Stub S. Serving their Country, Quietly and Bravely. 2016. http://www.thetower.org/article/serving-theircountry-quietly-and-bravely-bedouin-idf/.

[56] Tubi A, Feitelson E. Changing drought vulnerabilities of marginalized resource-dependent groups: a long-term perspective of Israel’s Negev Bedouin. Reg Environ Change. 2019;19(2):477–87. 10.1007/s10113-018-1420-9. 10.1007/s10113-018-1420-9

[57] WHO. (2022). World mental health report: transforming mental health for all. In: WHO Reports. 10.1136/bmj.o1593

[58] Wortmann JH, Jordan AH, Weathers FW, Resick PA, Dondanville KA, Hall-Clark B, Foa EB, Young-McCaughan S, Yarvis JS, Hembree EA, Mintz J, Peterson AL, Litz BT. Psychometric analysis of the PTSD checklist-5 (PCL-5) among treatment-seeking military service members. Psychol Assess. 2016. 10.1037/pas0000260. 26751087 10.1037/pas0000260

[59] Yahav R, Cohen M. Symptoms of acute stress in Jewish and Arab Israeli citizens during the second Lebanon war. Soc Psychiatry Psychiatr Epidemiol. 2007;42(10):830–6. 10.1007/S00127-007-0237-5/METRICS. 17668139 10.1007/S00127-007-0237-5/METRICS

[60] Yahel H. Rural or urban? Planning Bedouin settlements. Middle East Stud. 2021;57(4):606–24. 10.1080/00263206.2021.1887853. 10.1080/00263206.2021.1887853

[61] Yahel H, Abu-Ajaj A. Tribalism; religion; and state in Bedouin Society in the Negev: between preservation and change. Multidiscip J Natl Secur. 2021;24(2):54–71.

[62] Yip AM, Kephart G, Veugelers PJ. Individual and neighbourhood determinants of health care utilization: implications for health policy and resource allocation. Can J Public Health. 2002;93(4):303–7. 10.1007/bf03405022. 12154535 10.1007/bf03405022 PMC6980116

